# *Ulva* (*Enteromorpha*) Polysaccharides and Oligosaccharides: A Potential Functional Food Source from Green-Tide-Forming Macroalgae

**DOI:** 10.3390/md20030202

**Published:** 2022-03-10

**Authors:** Limin Ning, Zhong Yao, Benwei Zhu

**Affiliations:** 1 School of Medicine and Holistic Integrated Medicine, Nanjing University of Chinese Medicine, Nanjing 210023, China; ninglimin@njucm.edu.cn; 2 Laboratory of Marine Bioresource, College of Food Science and Light Industry, Nanjing Tech University, Nanjing 211816, China; yaozhong@njtech.edu.cn

**Keywords:** *Ulva*, polysaccharide, oligosaccharide, structure, preparation, activity

## Abstract

The high-valued utilization of *Ulva* (previously known as *Enteromorpha*) bioresources has drawn increasing attention due to the periodic blooms of world-wide green tide. The polysaccharide is the main functional component of *Ulva* and exhibits various physiological activities. The *Ulva* oligosaccharide as the degradation product of polysaccharide not only possesses some obvious activities, but also possesses excellent solubility and bioavailability. Both *Ulva* polysaccharides and oligosaccharides hold promising potential in the food industry as new functional foods or food additives. Studies on *Ulva* polysaccharides and oligosaccharides are increasing and have been the focus of the marine bioresources field. However, the comprehensive review of this topic is still rare and do not cover the recent advances of the structure, isolation, preparation, activity and applications of *Ulva* polysaccharides and oligosaccharides. This review systematically summarizes and discusses the recent advances of chemical composition, extraction, purification, structure, and activity of *Ulva* polysaccharides as well as oligosaccharides. In addition, the potential applications as new functional food and food additives have also been considered, and these will definitely expand the applications of *Ulva* oligosaccharides in the food and medical fields.

## 1. Introduction

**Figure 1:**
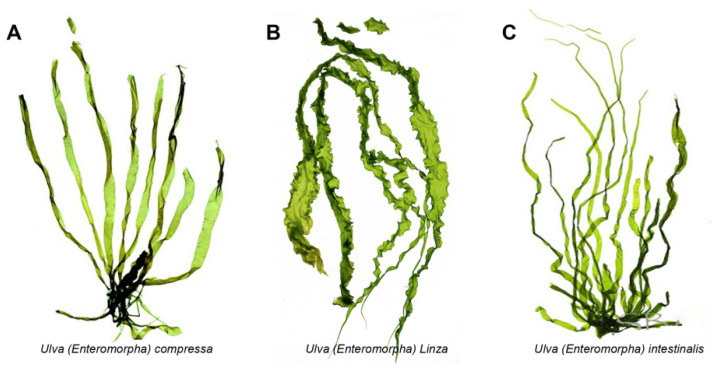
The morphology pictures of three kinds of *Ulva* species. (**A**). *Ulva compressa*; (**B**). *Ulva linza*; (**C**). *Ulva intestinalis*.

The *Ulva* (previously known as *Enteromorpha Enteromorpha*), known as green-tide-forming macroalgae, has drawn increasing attention in both the marine environment protection and marine bioresources fields [1,2]. Recently, the green tide blooms more and more frequently due to the global seawater eutrophication and temperature rise [3,4,5,6,7]. The largest *Ulva*-forming green tide in history occurred in the Yellow Sea of China this year, and covered almost 1746 km^2^, producing over 24 million tons of biomass [8]. The *Ulva* genus belongs to the Ulvaceae family and includes nearly 40 kinds of species such as *Ulva prolifera* (previously known as *Enteromorpha prolifera)*, *Ulva linza* (previously known as *Enteromorpha linza*), and *Ulva intestinalis* (previously known as *Enteromorpha intestinalis*) (as shown in Figure 1) [9]. For a long time, the *Ulva* and *Enteromorpha* were considered as two different genera, but the molecular evidence indicated that *Ulva* and *Enteromorpha* are not distinct evolutionary entities and should not be recognized as separate genera [9]. Therefore, the taxonomic name “*Enteromorpha*” is currently regarded as a synonym for *Ulva*.

The *Ulva* polysaccharide constitutes the main component of the cell wall of *Ulva* species algae, and it accounts for nearly 18% of the dry weight. In addition, it possesses various physiological properties such as antioxidant, anticoagulant, antitumor, antiaging and immune regulatory activities [10,11,12]. Therefore, the *Ulva* polysaccharides could be widely used as medicine and chemical agents in the agricultural and medical fields [13,14].

It is worth noting that another green algal polysaccharide, Ulvan, has also drawn increased attention, and its structure has been well characterized. The water-soluble sulfated polysaccharide is mainly extracted from *Ulva* sp. and consists of a linear backbone with L-rhamnose-3-sulfate (Rha3S), D-glucuronic acid (GlcUA), L-iduronic acid (IdoA) and D-xylose (Xyl), and the sulfate group is linked to the rhamnose. The two major repeating disaccharide units of ulvan are →4)-β-D-glucuronic acid (1→4)-α-L-rhamnose-3-sulfate (1→(A3S) and →4) α-L-iduronic acid (1→4)-α-L-rhamnose-3-sulfate (1→(B3S) [15]. However, the *Ulva* polysaccharide possesses a more complex chemical composition and fine structure. In addition, the *Ulva* polysaccharides exhibit great potential as functional foods and food additives due to their obvious metabolism-regulatory activity [16,17,18]. For instance, Guo et al. discovered that the polysaccharides extracted from *Ulva* *prolifera* could prevent high-fat diet-induced obesity in hamsters [19]. They also found that polysaccharides isolated from *Ulva* *prolifera* could protect against carbon tetrachloride-induced acute liver injury in mice via the activation of Nrf2/HO-1 signaling, and the suppression of oxidative stress, inflammation and apoptosis [20]. Li et al. found that the *Ulva* polysaccharides could improve blood glucose regulation, blood lipid metabolism and liver oxidative stress in T2DM cells [21]. However, the applications of the *Ulva* polysaccharide have been greatly limited by its poor solubility and low bioavailability [22]. In order to overcome this drawback, it is feasible to degrade the polysaccharide into oligosaccharide, which also possesses the biological activities but also has much better solubility and bioavailability [23]. The methods for polysaccharide degradation mainly include physicochemical or enzymatic methods [24,25,26]. In particular, the enzymatic method has drawn increasing attention due to its advantages, such as its mild reaction conditions and specific product distributions.

**Figure 2:**
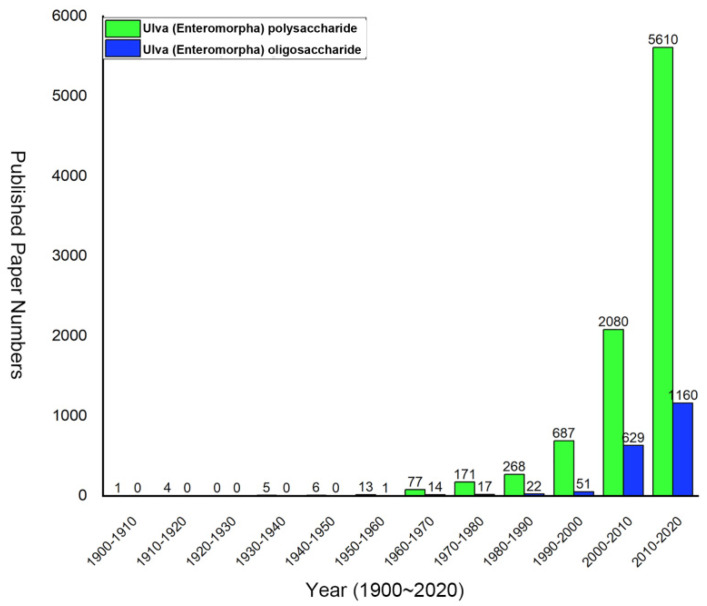
The numbers of published papers with keywords of *Ulva* polysaccharide and oligosaccharide between 1900 and 2020.

Studies of the *Ulva* polysaccharide and oligosaccharide have been increasing in the past two decades (Figure 2). Among these, most of them have focused on the structure and activity of the polysaccharides and oligosaccharides [14]. In addition, the preparation of oligosaccharides has drawn increasing attention [22]. However, there is not a comprehensive review which has summarized the recent advances in every aspect of the *Ulva* polysaccharide and oligosaccharide. In this review, we summarized and discussed the recent advances of chemical composition, extraction, purification, structure, activity and applications of *Ulva* polysaccharides as well as oligosaccharides. In addition, the potential applications as new functional foods and food additives have also been reviewed.

## 2. *Ulva* Polysaccharide

### 2.1. Chemical Composition and Structure of Ulva Polysaccharide

**Figure 3:**
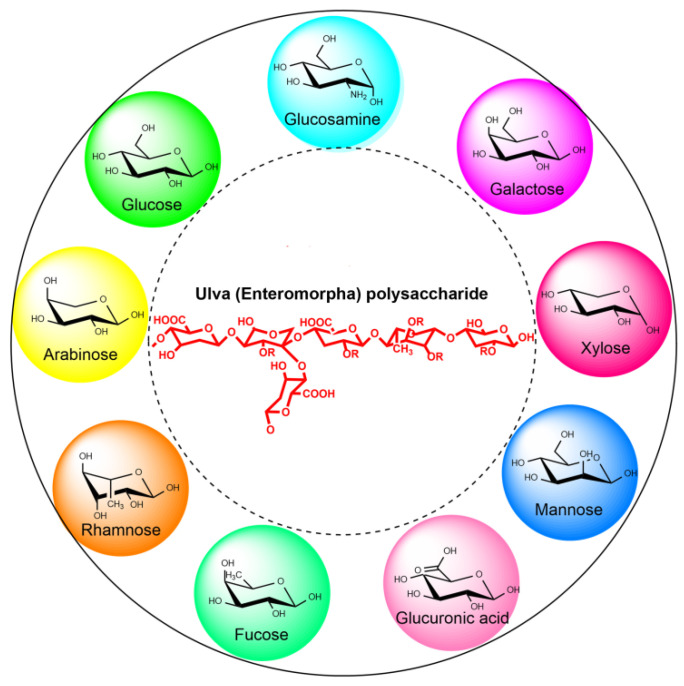
The main monosaccharide composition of *Ulva* polysaccharides.

The chemical composition of the *Ulva* polysaccharide is more complex than other common algal polysaccharides such as alginate (Phaeophyceae), carrageenan and agar (Rhodophyta) (Figure 3) [22,27,28,29]. The monosaccharide composition of *Ulva* polysaccharide mainly includes glucose, rhamnose, arabinose, xylose, mannose, galactose, fucose, glucosamine and glucuronic acid [27,28], which is different from algal polysaccharides (such as agar, carrageenan, alginate and fucoidan) that originate from brown and red algae. In addition, the chemical composition of *Ulva* polysaccharide differs in growth condition, harvesting season and the types of original *Ulva* species [14,30]. Qi et al. characterized the chemical compositions of the polysaccharides isolated from *Ulva linza*, *Ulva* *prolifera* and *Ulva clathrata*, respectively [28,31,32]. The results suggested that the chemical compositions of the three kinds of polysaccharides differed from each other. For instance, the polysaccharide from *Ulva* *linza* was composed of much rhamnose and a small amount of galactose, xylose and glucuronic acid; while the polysaccharides from *Ulva clathrata* contained larger amounts of arabinose and galactose and a small amount of rhamnose, fucose and xylose.

In addition, the main component (rhamnose) of the polysaccharides from *Ulva prolifera* is similar to the polysaccharide from *Ulva* *linza*. However, it contains some mannose, glucuronic acid and glucosamine, which is very different from the monosaccharide composition of the polysaccharide from *Ulva* *linza*. Moreover, the harvest time of *Ulva* algae also could influence on the chemical composition of the polysaccharide [31]. Shi et al. investigated the monosaccharide composition of *Ulva* polysaccharides, which isolated from the *Ulva clathrata* with different harvesting times [33]. The results suggested that the kinds of monosaccharide for polysaccharides isolated from the *Ulva clathrata* with different harvesting times seemed to be same. However, the ratios of these monosaccharides were different from each other. For instance, the monosaccharide (mannose, rhamnose, glucose, galactose and xylose) ratios of polysaccharides isolated from *Ulva clathrata* harvested in January and June are 6.74:65.56:5.54:2.83:19.33 and 2.81:67.55:2.31:2.71:24.61, respectively. In addition, the growth conditions also could exert an influence on the chemical composition of *Ulva* polysaccharide. Ji et al. analyzed the composition of *Ulva clathrata* samples and found that the monosaccharide compositions of *Ulva* *clathrata* under normal and explosive states exhibited obviously different levels [34]. The polysaccharides isolated from the *Ulva clathrata* under an explosive state contained iduronic acid, which did not exist in the polysaccharides isolated from *Ulva clathrata* under normal conditions.

The structure of polysaccharides that originate from *Ulva clathrata* is much more complex than other algal polysaccharides such as alginate, agar and carrageenan due to its complexity of monosaccharide composition, glycosidic linkage and group modification [14]. In addition, many factors such as the growth condition, harvesting season, the types of original *Ulva* species, etc., could lead to diverse structures of polysaccharides. Therefore, it is difficult to elucidate the fine structure of *Ulva* polysaccharide. Qi et al. investigated the fine structures of polysaccharides isolated from different sources in detail [28,32] (Table 1). The results indicated the polysaccharide of *Ulva linza* consisted of five fractions, namely MCS, MHS, SCS, SH1S and SH2S. In addition, it also included some fragments such as [→4)-β-D-Xylp-(1→], [→2)-α-L-Rhap-(1→], [→3)-α-L-Rhap-(1→], [→3, 4)-α-L-Rhap-(1→], [→2, 3)-α-L-Rhap-(1→], and [→2, 4)-α-L-Rhap-(1→]. The MCS and MHS fractions mainly contained [→4)-α-L-Rhap-(1→], [→2,4)-α-L-Rhap-(1→], [→4)-β-D-Xylp-(1→], [→4)-β-D-GlcAp-(1→], [→3)-α-L-Rhap-(1→] and [→2)-α-L-Rhap-(1→]. The SCS was consisted of [→3)-α-L-Rhap-(1→], [→2)-α-L-Rhap-(1→], [→4)-β-D-Xylp-(1→], [→4)-α-L-Rhap-(1→] and [→2,4)-α-L-Rhap-(1→], while the SH1S fraction composed of [→4)-α-L-Rhap-(1→], [→3)-α-L-Rhap-(1→], [→2,4)-α-L-Rhap-(1→], [→4)-β-D-Xylp-(1→] and [→4)-β-D-GlcAp-(1→]. The last fraction SH2S included [→4)-α-L-Rhap-(1→], [→4)-β-D-Xylp-(1→], [→2)-α-L-Rhap-(1→], [→3)-α-L-Rhap-(1→], [→3,4)-α-L-Rhap-(1→], [→2,3)-α-L-Rhap-(1→] and [→2,4)-α-L-Rhap-(1→]. They obtained four polysaccharide fractions (QC1S, QCQ2, QCQ3, and QHS) from *Ulva prolifera* and found that it mainly consisted of two disaccharide units, namely [→4)-β-D-GlcAp-(1→4)-α-L-Rhap3S-(1→] and [→4)-β-D-Xylp-(1→4)-α-L-Rhap3S-(1→]. The QC1S fraction contained [→4)-α-L-Rhap-(1→], [→4)-β-D-Xylp-(1→], [→2)-α-L-Rhap4S-(1→], [→3)-α-L-Rha4S-(1→], [→4)-α-L-Rhap2S-(1→] and [→3,4)-α-L-Rhap-(1→]. The QHS fraction mainly consisted of [→4)-α-L-Rhap-(1→], [→4)-β-D-Xylp-(1→], [→4)-β-D-GlcAp-(1→], [→3)-α-L-Rhap-(1→] and [→2,4)-α-L-Rhap-(1→]. In addition, three fractions (XCS, XH1S, XH2S) were isolated and characterized, the XCS fraction has been identified to have [→2)-β-D-Galp-(1→], [→3)-β-D-Galp-(1→], [→4)-β-D-Galp-(1→], [→6)-β-D-Galp-(1→], [→4)-β-L-Arap-(1→], [→2)-α-L-Rhap-(1→], [→3)-α-L-Rhap-(1→] and [→2)-α-L-Rhap-(1→]. Moreover, the XH1S fraction consisted of [→4)-β-L-Arap-(1→], [→3)-β-D-Galp-(1→], [→4)-β-D-Galp-(1→] and [→6)-β-D-Galp-(1→]. However, the XH2S fraction possessed new structures of [→4)-β-L-Arap3S-(1→], [→4)-β-L-Arap-(1→], [→3)-α-L-Rhap4S-(1→]. Jiao et al. characterized the fine structure of *Ulva intestinalis* polysaccharide and found that it mainly included the (1→)-Rha, (1→4)-Rha, (1→2, 4)-Rha, (1→) –Xyl, (1→2,3)-Xyl, (1→3)-Xyl, (1→4)-Glc and (1→3)-Gal structural units (Jiao et al., 2010; Jiao et al., 2009). They also determined the position of sulfate groups for different polysaccharide fractions and found that the position information is also very complicated and that there is not a uniform formula to describe the structures of *Ulva* polysaccharides [10,32,35].

### 2.2. Extraction and Purification of Ulva Polysaccharide

The extraction of the *Ulva* polysaccharide mainly used the common methods which were usually employed in the extraction of plant polysaccharides, especially the hot water extraction method [12,36]. The methods for extraction of *Ulva* polysaccharide have been summarized in Table 2. Xu et al. extracted the polysaccharide by incubation in hot water (90 °C) for 4 h and obtained 21.96% of polysaccharide [25]. Chattopadhyay et al. incubated the algal powder-water mixture at 80 °C for 1.5 h and extracted 18% of the crude polysaccharide [12]. The hot water extraction is the most commonly used method for preparation of plant polysaccharides. It could be operated easily and suitably for industrial scale application. However, the hot water extraction is time-consuming, and the extracted polysaccharides contained some soluble impurities. In order to improve the polysaccharide’s purity, alcohol was usually added remove the impurity before the hot water extraction. Wu et al. incubated the algal powder with 95% of alcohol at 80 °C for 2 h to remove the substance with low molecular weight and the purity reached 70% [37]. In addition, pH change also could remove the small molecules and further improve the polysaccharide’s purity. For instance, Sun et al. extracted the algal powder in 0.5 M of NaOH solution and incubated the mixture at 90 °C for 2 h. Finally, 33.3% of polysaccharide was obtained [38]. Song et al. incubated the sample in 0.05 M of HCl for 2 h and obtained 86.1% of soluble polysaccharide [39]. The addition of alcohol, acid or alkali could improve the purity of polysaccharide. Therefore, a combination of these agents could be tried in order to enhance the purity and improve the extraction efficiency. The ultrasonication could promote the dissolution of polysaccharide and has been widely used for extraction of *Ulva* polysaccharide [40]. Guo et al. extracted the polysaccharide under ultrasonication-treatment for 28 min and obtained 25.84 mg/g of crude polysaccharide [41]. Tang et al. obtained 17.42% of polysaccharide by incubation with ultrasonication of 531.17 W for 4.8 min [15]. However, the extraction efficiency of the ultrasonication method was not stable due to its short extraction time [42]. Furthermore, the microwave also has been used for extraction of *Ulva* polysaccharide due to its promotion of molecular motion [43]. Wang et al. used 610 W of microwave to assist the polysaccharide extraction and obtained 7.58% of crude polysaccharide [44]. Yuan et al. isolated the *Ulva* polysaccharide under 800 W of microwave and 95 °C, but only 4.04% of polysaccharide was obtained [45]. Therefore, microwave assistance could greatly reduce the extraction time, but could not promote the extraction yield and the purity of the polysaccharide [46,47]. The enzyme-assisted extraction method has drawn increasing attention due to its mild reaction condition and excellent extraction efficiency [48,49,50]. The protease and polysaccharide-degrading enzymes such as cellulase and pectin lyase could destroy the cell wall structure and promote the dissolution of polysaccharide [51]. Lü et al. added the protease into the extraction solution and obtained 27.75% of the polysaccharide [52]. Xu et al. used the cellulase to assist the extraction and the extraction ratio reached 20.22% [25]. However, a combination of different enzymes is needed to promote the extraction efficiency, and it also could increase the complexity of the reaction system. In conclusion, there are various methods for *Ulva* polysaccharide extraction and they all possess advantages and drawbacks. Therefore, it is reliable to combine these methods together to obtain the *Ulva* polysaccharide for further research. Because of the addition of enzymes, acid or alkali solution, the polysaccharide obtained by extraction needed to be purified for further structural characterization and activity investigation. At first, the protein could be removed by protease hydrolysis and Savage methods [40]. Then, the small substances produced by protein hydrolysis could be removed by dialysis. In order to obtain the purified polysaccharides, the ion exchange chromatography (IEC) and gel permeation chromatography (GPC) have usually been employed (Table 3) [53]. Qi et al. purified the *Ulva* polysaccharide from *Ulva* *linza* by Q Sepharose Fast Flow with NaCl as mobile phase and obtained five fractions [28,32]. Pan et al. purified four polysaccharide fractions from *Ulva intestinalis* by DEAE Sepharose Fast Flow with 0.5~1 M NaCl [54]. Jiao et al. used DEAE Sepharose CL-6B to purify the polysaccharide from *Ulva* *intestinalis*. In addition, the GPC has also been used for purification of *Ulva* polysaccharide [10]. Lü et al. purified two fractions by Sephadex G-100 with water as eluent [52] and Xu et al. used Sephadex G-75 to isolate the polysaccharide fractions by 1.0 mL·min^−1^ of H_2_O [25]. In practice, the ICE and GPC have usually been used together to purify the polysaccharide with higher purity. Lin et al. first separated the polysaccharide by DEAE-Cellulose 52 with 0.7 M NaCl and then the eluate was further purified by Bio-Gel P-2 with 0.85 mL·min^−1^ of H_2_O [55]. Tang et al. isolated the polysaccharide by DEAE-Sepharose CL-6B with 0.2~1.5 M NaCl and Sephadex G-200 with 0.85 mL·min^−1^ of H_2_O, respectively [11]. In addition, with the development of chemical engineering, more new technologies have been employed to purify the *Ulva* polysaccharide [56,57]. For instance, an integrated membrane separation process combining the tubular ceramic microfiltration (MF) membrane and the flat-sheet ultrafiltration (UF) membrane was developed to purify polysaccharides from *Ulva prolifera,* and the results suggested that the content of oligosaccharides reached 96.3% after purification by this integrated membrane separation process [56]. However, there is no report of polysaccharide purification on a large scale, and commercial polysaccharide is very expensive. It is essential to develop appropriate methods for adequate separation and purification of *Ulva* polysaccharide for commercial and industrial applications.

### 2.3. Activity of Ulva Polysaccharide

The activity of *Ulva* polysaccharide has been symmetrically investigated and characterized, and this green algal polysaccharide exhibited diverse biological activities such as antioxidant, antitumor, immunomodulatory, anticoagulant and hypolipidemic activities [11].

#### 2.3.1. Antioxidant Activity

Many algal polysaccharides such as alginate, carrageenan and agar possessed obvious antioxidant activity by cleaning the oxidant radicals and improving antioxidant enzymes’ activity [22]. Xu et al. evaluated the antioxidant activities of *Ulva* polysaccharide by determining their ability to scavenge 1, 1-diphenyl-2-picrylhydrazyl (DPPH), hydroxyl (OH^•^), and superoxide anion (O_2_^•−^) radicals [25]. The results suggested that the *Ulva* polysaccharide could clean up DPPH, OH^•^, and O_2_^•−^ [25]. It could also improve the activities of endogenous antioxidant enzymes such as catalase, glutathione peroxidase, and superoxide dismutase, which have been viewed as the major defense system against ROS during oxidative stress [40]. Moreover, Tang et al. found that the polysaccharides could reduce the content of maleic dialdehyde (MDA) in serum. The low MDA levels resulted in lower oxidant stress and lipid peroxidation [11].

#### 2.3.2. Antitumor Activity

The antitumor activity of *Ulva* polysaccharide has aroused increasing interest due to the tumor’s multiplicity worldwide [59,60]. Jiao et al. found that polysaccharides could inhibit tumor growth in S180 tumor-bearing mice, and could increase the relative spleen and thymus weight [10]. They also promoted the expression of tumor necrosis factor-alpha (TNF-α) in serum and induced lymphocyte proliferation, induced the production of TNF-α in macrophages, and stimulated macrophages to produce nitric oxide dose-dependently through the up-regulation of inducible NO synthase activity [35]. The *Ulva* polysaccharide could motivate modulation of the immune system to indirectly inhibit tumor cells without direct cytotoxicity [61].

#### 2.3.3. Immune Regulatory Activity

**Figure 4:**
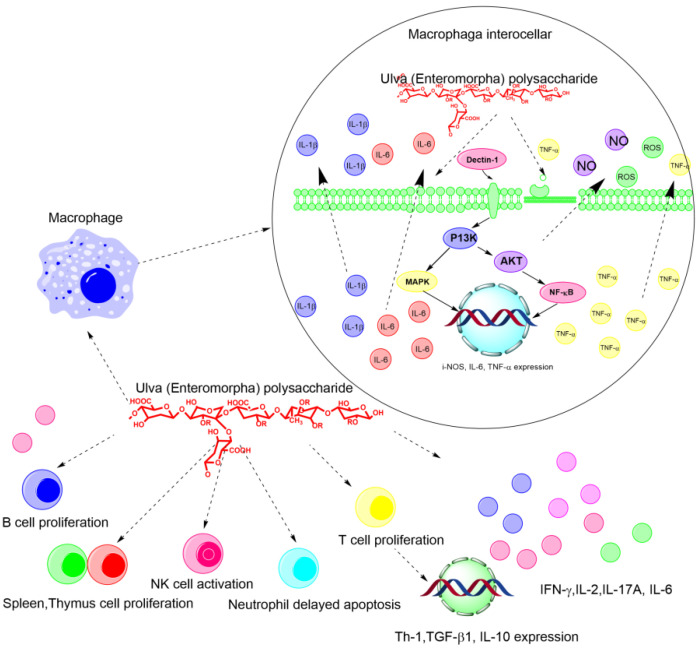
The schematic diagram of the immune regulatory and antitumor mechanism of *Ulva* polysaccharides on the molecular and cellular level.

The immune system includes nonspecific and specific immunity [62]. Nonspecific immunity can immediately respond to invaders without encountering previous pathogens, and gives signals to subsequently activate adaptive specific immunity [63]. Specific immunity involves B- and T-lymphocytes, and its function is activated immediately after the initial antigenic stimulus [64]. The *Ulva* polysaccharide can significantly increase the relative spleen and thymus weight of tumor-bearing animals, promote the secretion of tumor necrosis factor alpha (TNF-α), stimulate lymphocyte proliferation, and augment phagocytosis and secretion of NO and TNF-α in peritoneal macrophages [65]. In addition, the *Ulva* polysaccharide could promote the proliferation of B lymphocytes and T lymphocytes, activate the NK cell and induce the delayed apoptosis of neutrophils, as shown in Figure 4. More specifically, the polysaccharides could increase the production of reactive oxygen species (ROS), IL-6, and TNF-α through regulating the expressions of iNOS, IL-6, and TNF-α. In addition, the polysaccharides can strengthen the macrophage phagocytic activity, activate NK cells, increase thymus and spleen indices, and delay neutrophil apoptosis [7,66] (Figure 4).

#### 2.3.4. Anticoagulant Activity

It has been reported that polysaccharides from green alga have been investigated, showing stronger anticoagulant activities than those from brown and red alga [28,67,68]. Wang et al. investigated and elucidated the anticoagulant activity of polysaccharide from green algae *Ulva linza* in the coagulation assays, and activated partial thromboplastin time (APTT), thrombin time (TT) and prothrombin time (PT) [69]. The results suggested that the sulfated polysaccharides could prolong APTT and TT, but not TP. These activities strongly depended on the degree of sulfation (DS), the molecular weights (MW) and the branching structure of polysaccharides [69]. Qi et al. evaluated the anticoagulant activity of polysaccharides from *Ulva clathrata* and an in vitro anticoagulant assay indicated that FEP effectively prolonged the activated partial thromboplastin time and thrombin time [28,32].

#### 2.3.5. Hypolipidemic Activity

Hyperlipidemia, as a common endocrine disease, induces cerebrovascular and cardiovascular activity and atherosclerosis [70,71]. While hypolipidemic drugs such as statins prevent and cure hyperlipidemia, their side effects cannot be ignored [70]. Teng et al. reported that *Ulva prolifera* polysaccharides presented high anti-hyperlipidemic activities which inhibited the body weight gain and also decreased triacylglycerol (TG), the total cholesterol (TC), and low-density lipoprotein cholesterol (LDL-C) levels of plasma and liver [72]. They also inhibited the expressions of sterol regulatory element-binding protein-1c (SREBP-1c) and hepatic acetyl-CoA carboxylase (ACC) in high-fat diet rats. SREBP-1c enhances the transcription of the required genes for fatty acid synthesis [72]. ACC, as the rate-limiting enzyme in de-novo lipogenesis, controls the β-oxidation of fatty acids in the mitochondria. Moreover, *Ulva prolifera* polysaccharides showed pancreatic lipase inhibition activity [45]. The polysaccharide from *Ulva prolifera* exhibited a stronger hypolipidemic effect than simvastatin and enhanced endogenous antioxidant enzymes and decreased MDA content and lipid peroxidation in serum [72,73].

## 3. Ulva Oligosaccharides

The *Ulva* polysaccharide which acted as the main component of *Ulva* sp. has attracted increasing attention in the algal bioresources field. As discussed above, the reports of extraction, isolation, purification, structural characterization and physiological activity of this polysaccharide have increased year after year. In addition, the techniques for extracting the polysaccharide have developed rapidly on an industrial scale, and this advance established a solid foundation for the wide application of *Ulva* polysaccharides. However, the applications of polysaccharide for the food and medical industries have been greatly restricted by its high molecular weight and low solubility, so the degradation of polysaccharide into oligosaccharides has been the area of emerging focus in utilization of *Ulva* polysaccharide bioresources.

### 3.1. Preparation of Ulva Oligosaccharides

The *Ulva* oligosaccharides retained the versatile activities of polysaccharide and were found to possess excellent solubility and bioavailability, and they have drawn increasing attention from scientists from the food and medical fields [23]. The preparation of *Ulva* oligosaccharides mainly depended on physical degradation, chemical hydrolysis and enzymatic preparation, as shown in Table 4 [22]. Similar to the preparation of other algal oligosaccharides such as alginate oligosaccharides and carrageenan oligosaccharides, the microwave-assisted method has been widely used. Li et al. prepared *Ulva* oligosaccharides by a microwave-assisted acid hydrolysis method and the results showed that only glycosidic linkages were left without breaking significant structural units [74]. Duan et al. degraded *Ulva* polysaccharide with HCl and assisted by microwave. The optimal degradation conditions were 900 W at 50 °C for 10 min with 5% H_2_O_2_ in 1 mol/L hydrochloric acid solution by single factor and orthogonal experiments [75]. Zhang et al. used the ascorbic acid and H_2_O_2_ as degradation reagents to degrade the polysaccharides in order to obtain the lower molecular weight products. The enzymatic preparation of *Ulva* oligosaccharides has been the research focus due to its advantages such as mild reaction conditions, specific products’ distribution, etc [26].

Zhang et al. degraded the *Ulva* polysaccharide by degrading enzymes produced by *Alteromonas* sp. A321 and the oligosaccharides yield reached 61.21% [77]. Xu et al. degraded *Ulva* polysaccharide by the addition of pectin lyase (9.6 U/mL) and obtained oligosaccharides with different molecular weights [25]. Surprisingly, there are no reports of the gene information for specific enzymes which could degrade the *Ulva* polysaccharide. As we know, other algal polysaccharides such as alginate, agar, and carrageenan could be specifically degraded by the respective enzymes, namely alginate lyases, agarases and carrageenases. Li et al. screened and identified a new *Ulva* polysaccharide-degrading strain *Alteromonas* sp. A321 from the rotten green algae [76,78]. They characterized the enzymes produced by *Alteromonas* sp. A321 and sequenced the N-terminal of them [76,78]. However, it was unable to be determined whether the two enzymes belonged to polysaccharide lyase or glycosidic hydrolase. Therefore, further and systematic research is required to investigate and elucidate this question.

### 3.2. Separation and Purification of Ulva Oligosaccharides

The methods used for separation and purification of *Ulva* oligosaccharides are similar with the methods for purification of *Ulva* polysaccharides. Xu et al. sequentially purified the *Ulva* oligosaccharides by DEAE Cellulose-52 chromatography and Sephadex G-100 chromatography. In addition, the molecular weights of these three fractions were measured to be 103, 45.4, and 9.8 kDa, respectively, using high performance gel permeation chromatography (HPGPC) [25]. Lü et al. purified the degraded polysaccharide by Sephadex G-100 with water as eluent, and obtained a yield of 40.00% [52]. Li et al. employed a TSK G4000-PWxl column, using 0.05 M NaNO_3_ aqueous solution as the mobile phase at a flow rate of 0.5 mL/min with a column temperature of 30 °C to purify the degraded *Ulva* [76]. According to the examples discussed above, the purification methods of *Ulva* oligosaccharides seemed similar to the polysaccharides purification procedure, but the purpose of polysaccharide purification is to remove the impurities from the polysaccharide system, while the target of oligosaccharide purification is to separate the oligosaccharide fraction from the mixture. Therefore, it is more difficult to obtain the oligosaccharide monomer since the methods for polysaccharides’ purification have developed, and it is promising for researchers to find more suitable methods for purification of *Ulva* oligosaccharides.

### 3.3. Activity of Ulva Oligosaccharides

So far, more and more studies are focusing on the activity of *Ulva* oligosaccharides [22,23]. However, the current reports are still very scattered with the mechanism of related activity and the structural-activity relationship of oligosaccharides was still undefined due to the complexity of the *Ulva* oligosaccharides’ structure. Lü et al. evaluated the antibacterial activity of *Ulva* oligosaccharides and their selenized derivatives prepared by acid method [52]. They found that the selenized *Ulva* oligosaccharides showed stronger inhibitory activity towards *Eschetichia coli* and plant pathogenic fungi than that to *Staphylococcus aureus* [52,79]. Liu et al. studied the anti-aging and anti-oxidation effects of *Ulva* oligosaccharides in SAMP8 mice [23]. They found that *Ulva* oligosaccharides can protect neurons in the hippocampus by significantly reducing the secretion of inflammatory factors such as IFN-γ, TNF-α and IL-6, and improving the brain-derived neurotrophic factor (BDNF) [23]. Liu et al. evaluated the immunoregulatory effect of *Ulva* oligosaccharides in a cyclophosphamide-induced immunosuppression mouse model [80]. It can be found that *Ulva* oligosaccharides can activate the immune system by promoting the secretion of NO, up-regulating the expression of cytokines such as IL-1β, IL-6 and TNF-α, and activating inflammatory bodies such as iNOS, COX2 and NLRP3 [80]. Xu et al. studied the antioxidant activities of three *Ulva* oligosaccharides, and found that *Ulva* oligosaccharides can effectively eliminate the DPPH, OH^•^, and O_2_^•−^ [25]. Li et al. investigated the antioxidant capacity of *Ulva* oligosaccharides and found that the activity was closely related to molecular weight [74]. Specifically, *Ulva* oligosaccharides with low molecular weight can scavenge superoxide anion and hydroxyl radicals with an IC50 of 0.39 mg/mL [74]. Zhang et al. found that 2.28 mg/mL of *Ulva* oligosaccharides prepared by an H_2_O_2_ oxidation method can scavenge 92.2% of the hydroxyl radical, which is higher than *Ulva* polysaccharides with the same concentration [26]. That is probably because there were more hydroxyl groups in the oligosaccharides’ structure [26]. Cui et al. prepared complexes of Fe^2+^ ions and *Ulva* oligosaccharides, which can be used to treat iron deficiency anemia as a nutritional supplement for iron [81]. Wang et al. discovered that *Ulva* oligosaccharides possessed an anticoagulant activity which was closely related to the number and distribution of sulfuric acid groups in oligosaccharides [69]. Jin et al. prepared ep-3-H, a glucuronic-xylo-rhamnose-component, from *Ulva* prolifera, and found that EP-3-H could inhibit cell proliferation of human lung cancer cells by interacting with the fibroblast growth factors FGF1 and FGF2 [82]. In addition, the physiological activities may differ in *Ulva* oligosaccharides and polysaccharides, but the specific mechanism still remains unclear. However, we could propose the possible reasons based on some experience. The active groups appeared after the linkage of the polysaccharide was broken down by physical, chemical or enzymatic hydrolysis, and therefore the activities of *Ulva* oligosaccharide became more obvious than the polysaccharide. To sum up, the current studies on the activity of *Ulva* oligosaccharides are relatively superficial since there is still no appropriate method to obtain oligosaccharides with a fine structure for studying the structure-activity relationship of oligosaccharides due to their quite complex structure.

## 4. Conclusions and Future Perspective

**Figure 5:**
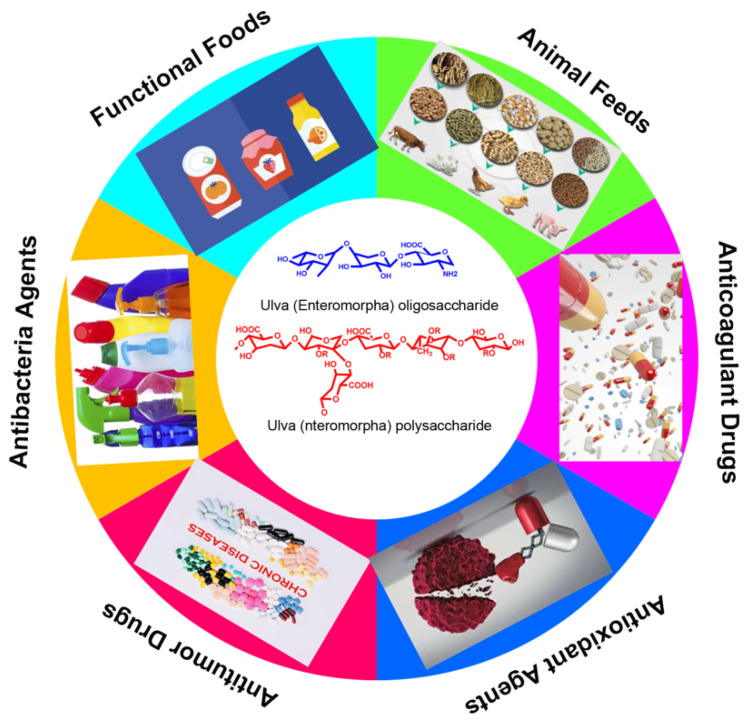
The potential and promising applications of *Ulva* polysaccharide and oligosaccharides.

In recent years, the biomass of *Ulva* has increased rapidly worldwide, resulting in a large number of green tides [4,80,83]. Actually, *Ulva prolifera* has invaded the Yellow Sea for 15 consecutively years, which has damaged the marine ecological environment in Qingdao and the coastal cities of Shandong Province. It is therefore urgent to effectively curb the growth of *Ulva prolifera* and achieve the harmless and high-value utilization of *Enteromorpha prolifera* [84,85,86] (as shown in Figure 5). For instance, the *Ulva* polysaccharide and oligosaccharide could eliminate the oxidative radicals such as DPPH, OH^•^, and O_2_^•−^, and they could also promote the proliferation of probiotics of intestinal microbiome composition. In addition, the *Ulva* polysaccharide exhibited obvious hypolipidemic activity; therefore, the *Ulva* polysaccharide and oligosaccharide can be used as a functional food, a food additive, an antioxidant agent, and animal feed. Due to its excellent rheological properties, gelling behavior, texture characteristics and antibacterial activity, the *Ulva* polysaccharide could be developed as a novel medical dressing to prevent bacterial infection. More importantly, the *Ulva* polysaccharide and oligosaccharide both possess obvious physiological activities such as immune regulatory, antitumor, anticoagulant and hypolipidemic activities, they are important resources for developing novel marine drugs for curing various malignant tumors, and they are used in the treatment of hyperlipidemia, hypertension and other metabolic diseases. This kind of carbohydrate that originated from green algae has drawn increasing attentions and became a topic of much discussion in the marine bioresources and functional foods fields.

*Ulva* polysaccharide, as the main active ingredient of the *Ulva* species, can be used to develop new foods, medicine and health care products. At present, it is easy to extract *Ulva* polysaccharides at a large-scale level. Nevertheless, it still cannot meet the requirements of high-value utilization due to its low purity. So it is now one of the areas of intensive research to find out how to prepare *Ulva* polysaccharides with high purity. On the other hand, the solubility and bioavailability of *Ulva* polysaccharides is restrained by their high molecular weight (>400 kDa), which has further limited the biological activity and application of *Ulva* polysaccharides. It is therefore another area of focus to prepare *Ulva* oligosaccharides with *Ulva* polysaccharides degrading enzymes. In addition, it is still a great challenge to analyze the structure of *Ulva* polysaccharides and *Ulva* oligosaccharides due to the complex and diverse monosaccharide composition and glycosidic bond connection modes in *Ulva* polysaccharides. Therefore, it is of great significance to accurately analyze the fine structure of *Ulva* polysaccharides and oligosaccharides, which will promote the study of the structure-activity relationship and the high value utilization of *Ulva* polysaccharides and oligosaccharides. In addition there is a long history of humans utilizing the *Ulva* bioresources for food, and some beneficial foods such as biscuits, noodles and vegetarian meatballs have been developed. However, the applications of *Ulva* polysaccharide and oligosaccharide as functional foods have not been realized until now because the studies of the activity and function of the *Ulva* polysaccharide and oligosaccharide are ongoing. We believe that the *Ulva* polysaccharide and oligosaccharide could be used as a functional food such as the alginate oligosaccharides in the near future when we have obtained an adequate understanding of them.

## Figures and Tables

**Table 1:** The summary of structures of polysaccharides originated from different species.

*Ulva linza*				*Ulva prolifera*				*Ulva clathrata*			
Fraction	MCS	Main components	[→4)-α-L-Rhap-(1→] [→2,4)-α-L-Rhap-(1→] [→4)-β-D-Xylp-(1→] [→4)-β-D-GlcUAp-(1→]	Fraction	QC1S	Main components	[→4)-α-L-Rhap-(1→] [→4)-β-D-Xylp-(1→]	Fraction	XCS	Main components	[→2)-β-D-Galp-(1→] [→3)-β-D-Galp-(11→] [→4)-β-D-Galp-(1→] [→6)-β-D-Galp-(1→]
		Other components	[→3)-α-L-Rhap-(1→] [→2)-α-L-Rhap-(1→]			Other components	[→2)-α-L-Rhap4S-(1→] [→3)-α-L-Rha4S-(1→] [→4)-α-L-Rhap2S-(1→] [→3,4)-α-L-Rhap-(1→]			Other components	[→4)-β-L-Arap-(1→] [→2)-α-L-Rhap-(1→] [→3)-α-L-Rhap-(1→] [→2)-α-L-Rhap-(1→]
		Sulfated position	The C_3_ of [→4)-α-L-Rhap-(1→]							Sulfated position	The C_6_ or C_2_ [→4)-β-D-Galp-(1→], the C_4_ or C_2_ of [→6)-β-D-Galp-(1→]
	MHS	Main components	[→4)-α-L-Rhap-(1→] [→2,4)-α-L-Rhap-(1→] [→4)-β-D-Xylp-(1→] [→4)-β-D-GlcUAp-(1→]		QHS	Main components	[→4)-α-L-Rhap-(1→] [→4)-β-D-Xylp-(1→] [→4)-β-D-Glc UAp-(1→]		XH1S	Main components	[→4)-β-L-Arap-(1→]
		Other components	[→3)-α-L-Rhap-(1→] [→2)-α-L-Rhap-(1→]			Other components	[→3)-α-L-Rhap-(1→] [→2,4)-α-L-Rhap-(1→]			Other components	[→3)-β-D-Galp-(1→] [→4)-β-D-Galp-(1→] [→6)-β-D-Galp-(1→]
		Sulfated position	The C_3_ of [→4)-α-L-Rhap-(1→]			Sulfated position	The C_3_ of [→4)-α-L-Rhap-(1→]			Sulfated position	The C_3_ of [→4)-β-L-Arap-(1→]
	SCS	Main components	[→3)-α-L-Rhap-(1→] [→2)-α-L-Rhap-(1→] [→4)-β-D-Xylp-(1→] [→4)-α-L-Rhap-(1→] [→2,4)-α-L-Rhap-(1→]		QCQ2	-			XH2S	Main components	[→4)-β-L-Arap3S-(1→]
		Sulfated position	The C_2_ or C_4_ of [→3)-α-L-Rhap-(1→], the C_3_ or C_4_ [→2)-α-L-Rhap-(1→], the C_2_ of [→4)-α-L-Rhap-(1→]							Other components	[→4)-β-L-Arap-(1→] [→3)-α-L-Rhap4S-(1→]
	SH1S	Main components	[→4)-α-L-Rhap-(1→] [→3)-α-L-Rhap-(1→] [→2,4)-α-L-Rhap-(1→] [→4)-β-D-Xylp-(1→] [→4)-β-D-Glc UAp-(1→]		QCQ3	-					
		Sulfated position	The C_3_ of [→4)-α-L-Rhap-(1→]								
	SH2S	Main components	[→4)-α-L-Rhap-(1→] [→4)-β-D-Xylp-(1→]								
		Other components	[→2)-α-L-Rhap-(1→] [→3)-α-L-Rhap-(1→] [→3,4)-α-L-Rhap-(1→] [→2,3)-α-L-Rhap-(1→] [→2,4)-α-L-Rhap-(1→]								
		Sulfated position	The C_3_ of [→4)-α-L-Rhap-(1→]								
	[→4)-β-D-Xylp-(1→]										
Other components	[→2)-α-L-Rhap-(1→] [→3)-α-L-Rhap-(1→] [→3,4)-α-L-Rhap-(1→] [→2,3)-α-L-Rhap-(1→] [→2,4)-α-L-Rhap-(1→]										

**Table 2:** The summary of extraction of *Ulva* polysaccharide.

Extraction Method	Procedure Time	Yield	Recovery	Reference
Hot water extraction with Hot water (90 °C)	4 h	21.96%	-	[25]
Hot water extraction with Hot water (80 °C)	1.5 h	18%	-	[12]
Hot solution extraction with 95% of alcohol (80 °C)	2 h	-	70%	[37]
Hot alkaline solution extraction with 0.5 M NaOH (90 °C)	2 h	33.3%	-	[38]
Acidic solution extraction with 0.05 M HCl	2 h	86.1%		[39]
Ultrasonication treatment Ultrasonication treatment	28 min	25.84%	-	[41]
Ultrasonication treatment Ultrasonication (531.17 W)	4.8 min	17.42%	-	[15]
Ultrasonication treatment Ultrasonication (610 W)	-	-	7.58%	[44]
Ultrasonication treatment Ultrasonication (610 W)	-	-	4.04%	[45]
Enzymatic extraction with Protease	-	27.75%	-	[52]
Enzymatic extraction with Cellulase	-	20.22%	-	[25]

**Table 3:** The summary of purification of *Ulva* polysaccharide.

Purification Method	Column	Mobile Phase	Speed	Reference
IEC	Q Sepharose Fast Flow	0~2 M NaCl	0.5~2 mL/min	[31]
IEC	DEAE Sepharose Fast Flow	0~2 M NaCl	0.92 mL/min	[33]
IEC	DEAE Sepharose CL-6B	0.9%NaCl	0.18 mL/min	[35]
IEC	DEAE Cellulose 52	0.2~0.8 M NaCl	0.5 mL/min	[25]
IEC	DEAE Sephadex A-25	0~4 M NaCl	0.5 mL/min	[58]
GPC	Sephadex G-75	H_2_O	1.0 mL/min	[25]
GPC	Sephadex G-100	H_2_O	0.4 mL/min	[52]
GPC	SephacryTm S-300 HR	0.9% NaCl	0.5 mL/min	[58]
GPC	Sephacryl S-300 HR	0.2 M NH_4_HCO_3_	0.5 mL/min	[30]
GPC	Sephacryl S-400/HR	0.2 M NH_4_HCO_3_	0.3 mL/min	[28]
IEC+GPC	DEAE Cellulose 52, Bio-Gel P-2	0.7 M NaCl	0.85 mL/min	[40]
IEC+GPC	DEAE-Sepharose CL-6B, Sephadex G-200	0.2~1.5 M NaCl	0.8 mL/min	[11]

**Table 4:** The summary of methods for preparation of *Ulva* oligosaccharides.

Preparation Method	Structure	Molecular Weight	Bioactivities	Reference
Microwave-assisted acid hydrolysis	-	3.1 kDa	Antioxidant activity	[76]
Microwave-assisted acid hydrolysis	-	53.59 kDa	Antioxidant activity	[75]
H_2_O_2_ degradation	-	-	Antioxidant activity	[26]
Enzymatic degradation	-	243, 341, 401, 503, 665 Da	-	[77]
Enzymatic degradation	-	103, 45.4, 9.8 kDa	Antioxidant activity	[25]
Enzymatic degradation	Rha_1_(SO_3_H)_1_, Rha_1_(SO_3_H)_1_Glc_1_, Rha_2_(SO_3_H)_2_Glc_1_, Rha_3_(SO_3_H)_3_Glc_1_Xyl_1_	244, 402, 628, 760 Da	-	[78]

## Data Availability

Not applicable.
